# Hypercalcemia in a patient with cholangiocarcinoma: a case report

**DOI:** 10.1186/1755-7682-2-35

**Published:** 2009-10-30

**Authors:** Ioannis D Xynos, Stavros Sougioultzis, Athanasios Zilos, Konstantinos Evangelou, Gregorios S Hatzis

**Affiliations:** 1 Department of Pathophysiology, National and Kapodistrian University of Athens, Medical School, Laikon General Hospital, Greece; 2 Department of Pathology, National and Kapodistrian University of Athens, Medical School, Greece

## Abstract

**Background:**

Humoral hypercalcemia of malignancy is rarely associated with cholangiocarcinoma (CC).

**Case report:**

A 77-year-old man was admitted with confusion. Computer tomography showed a large multinodular mass in the right lobe of the liver and smaller lesions in the right lung. Liver histology confirmed the diagnosis of CC. Elevated calcium levels and suppressed intact parathyroid hormone in the absence of skeletal metastases or parathyroid gland pathology suggested the diagnosis of humoral hypercalcemia of malignancy (HHM). Treatment of hypercalcemia with saline infusion, loop diuretics, biphosphonate and calcitonin was effective in normalizing calcium levels and consciousness state within 48 hours, but a relapse occurred 4 weeks later and the patient succumbed to his disease.

**Conclusion:**

Clinicians should be aware of this rare manifestation of CC as prompt and aggressive correction of hypercalcemia alleviates symptoms and improves patient's quality of life, despite the poor overall prognosis.

## Background

Hypercalcemia occurs in around 30% of patients with malignant disease. It is caused either by tumor production of humoral factors [humoral hypercalcemia of malignancy (HHM)] or by locally enhanced bone resorption associated with metastatic lesions of solid cancers [1]. Cholangiocarcinoma (CC) is an epithelial tumor of the biliary tree that accounts for 10 to 15% of all hepatobiliary malignancies. It represents 3% of gastrointestinal tract cancers and its incidence is increased worldwide [2]. The majority of patients with CC are older than 65 years of age and although cases of long-term survival have been reported after resection, most patients with unresectable disease die between 6 months and 1 year following diagnosis [3]. HHM has been rarely documented in patients with CC. In this report, we present a case of advanced CC associated with clinical and laboratory findings consistent with HHM.

## Case presentation

**Figure 1:**
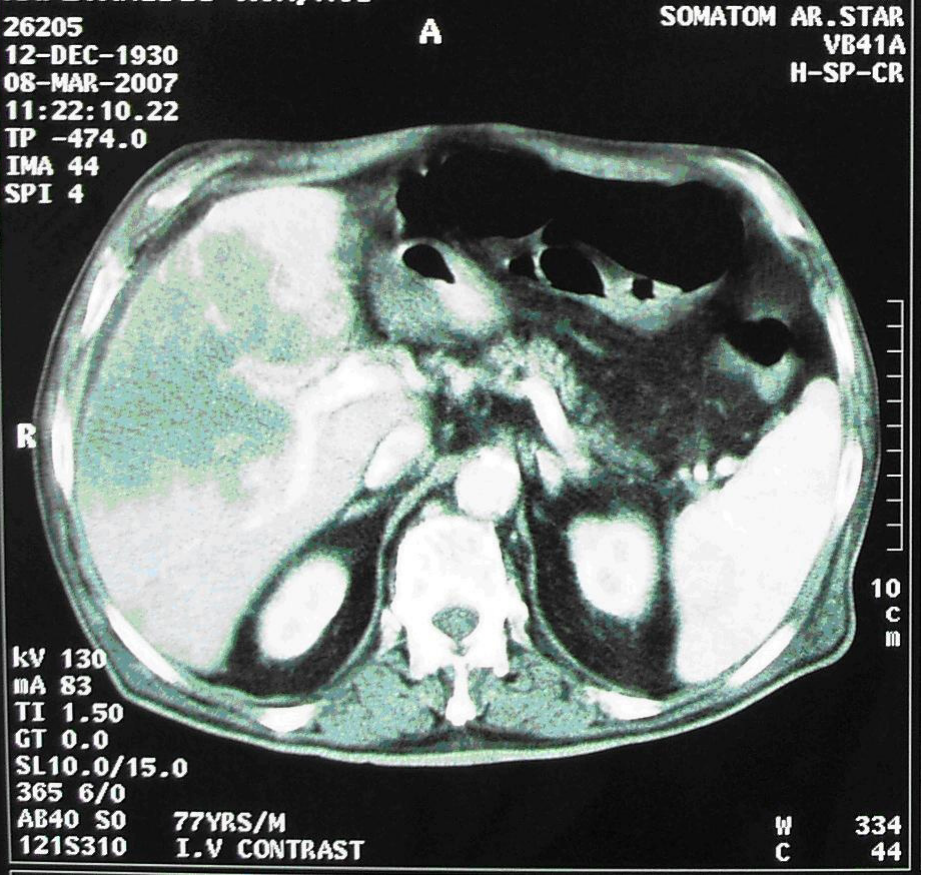
**Computer tomography image of the abdomen showing a lobulated mass in the right lobe of the liver**.

**Figure 2:**
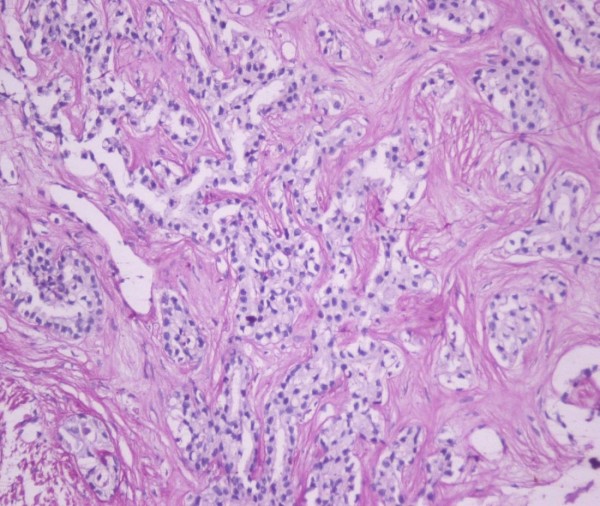
**Arborizing tubular structures lined by columnar neoplastic cells within dense collagenous stroma**. (H&E counterstain, magnification: ×200).

A 77-year-old Caucasian man with a history of type II diabetes and hypertension presented with confusion. Other symptoms included general fatigue, anorexia, weight loss, nausea and occasional vomiting that extended over a period of 2 months. He was afebrile and physical examination revealed an enlarged non-tender liver with an irregular border. Blood tests showed a white blood cell count of 14470 K/μl, Neu 82%, urea 100 mg/dl (normal range, 17-50), creatinine 1.4 mg/dl (normal range, 0.7-1.4), calcium 12.6 mg/dl (normal range, 8.6-10.2), phosphorus 2.9 mg/dl (normal range, 2.7-4.5), albumin 3.6 g/dl (normal range, 3.5-5.5), aspartate aminotransferase (SGOT) 77 U/L (normal range, 5-40), alanine aminotransferase (SGPT) 49 U/L (normal range, 5-40), alkaline phosphatase (ALP) 563 U/L (normal range, 64-280), gamma-glutamyl transferase (γGT) 500 U/L (normal range, 11-49), and plasma ammonia 44 μg/dl (normal range, < 75). The rest of blood routine biochemistry was unremarkable. Parathyroid hormone (PTH) was suppressed at 1.55 pg/dl (normal range, 8-76) and carcinoembryonic antigen 19-9 (CA 19-9) was elevated at 223 U/ml (normal range, <37). Computer tomography (CT) revealed a large multinodular mass in the right lobe of the liver consistent with neoplastic disease (Figure 1) and smaller nodules in the right lung. Brain CT was normal, bone scan with ^99m^Tc-MDP showed no evidence of metastatic bone disease, and parathyroid scan with ^99m^Tc-MIBI double phase was unremarkable. A liver surgical biopsy confirmed the diagnosis of CC (Figure 2). Neoplastic cells stained positively for cytokeratin 7 and 19.

The patient was treated with intravenous hydration, furosemide, ibandronate, and calcitonin, with calcium levels and consciousness state normalizing within 48 hours. He declined chemotherapy and was discharged home, from where he was readmitted 4 weeks later with relapsed hypercalcemia and progressively worsening confusion. A repeat CT scan showed significant expansion of the liver mass. Treatment of hypercalcemia resulted again in rapid restoration of his consciousness level. However, overall prognosis was poor and the patient died due to progressive disease within 3 months of diagnosis.

## Discussion

HHM is typically associated with squamous cell carcinomas of head and neck, esophagus and lung. Other tumors commonly associated with HHM include breast, renal, bladder and ovarian cancers, human T-cell lymphotropic virus-1 lymphoma, and some endocrine tumors. It is rarely seen in association with colon adenocarcinoma, gastric carcinoma, small cell carcinoma, and prostate cancer. HHM is characterized biochemically by elevated serum calcium, low serum phosphorous, low PTH, low 1,25 (OH)2 vitamin D levels and elevated nephrogenous cyclic AMP excretion rate [4].

CC is rarely associated with HHM. A retrospective analysis of 190 CC cases with hypecalcemia by Oldenburg et al. showed that in 17.5% of those cases hypercalcaemia was not associated with metastatic bone disease, while 5 patients had serum immunoreactive PTH levels consistent with ectopic hyperparathyroidism [5]. Since then, a few case reports in the english literature have linked CC with HHM [6-10].

Many factors including vascular endothelial growth factor (VEGF) and interleukin -8 and -11 have been implicated in promoting HHM, although currently, parathyroid hormone-related protein (PTH-rP) is believed to be the major mediator [11]. Circulating levels of PTH-rP are elevated in 80% of patients with HHM [4]. PTH-rP shares many structural features with PTH and both share the same PTH receptor [12,13]. Similarly to PTH, PTH-rP interacts with the PTH/PTH-rP receptor that mediates the renal tubular reabsorption of calcium and stimulates osteoclastic bone resorption resulting in hypercalcemia [14]. Hence, medical treatment of HHM should include hydration by saline infusion, loop diuretics to promote urinary calcium excretion, and antiresorptives such as calcitonin and biphosphonates [15].

## Conclusion

We presented a case of advanced CC associated with symptomatic hypercalcemia. Increased calcium levels and suppressed intact PTH levels, in the absence of metastatic bone disease and parathyroid gland pathology, suggested the diagnosis of HHM. Clinicians should be aware of this rare manifestation of CC since prompt correction of hypercalcemia affords symptomatic relief and improves the quality of life of patients.

## Consent

Written informed consent was obtained from the patient's next of kin (after death) for publication of this case report and any accompanying images. A copy of the written consent is available for review by the Editor-in-Chief of this journal.

## Competing interests

The authors declare that they have no competing interests.

## Authors' contributions

IDX, SS, AZ, GSH were involved in the direct care of this patient. In addition IDX and GSH drafted the manuscript. KE performed the histology on the liver biopsy. All authors have read and approved the manuscript.
